# 

**Published:** 1915-07

**Authors:** 


### ANNOUNCEMENTS

### PANAMA-PACIFIC DENTAL CONGRESS.

August 30th, the date on which the Panama-Pacific Dental Congress will meet in San Francisco, is near at hand. The Committee of Organization desires to announce that the Congress will open on time with an exceptionally good program. About 130 papers and over 200 clinics are now promised for the program, the resume of which follows.

Practically all the exhibit space at the disposal of the committee is now occupied and no other Congress has had such a complete exposition of dental and pharmaceutical goods as will be presented here. Everything points to a large and successful meeting. Nearly eleven hundred applications for memberships are now on file and more are coming in daily.

The Membership Committee urges all who expect to attend the Congress, to fill out their application blanks, have them signed by a member of the Executive Committee of the state in which they reside, and forward with check, draft or P. O. Money Order for Ten Dollars to the Secretary, Dr. A. M. Flood, 240 Stockton St., San Francisco. This should be done as soon as possible as it will save the dentist considerable trouble and annoyance, and will facilitate the work of registration. Those who have not paid for their membership, nor have filed their applications for membership, properly endorsed, but expect to obtain membership in the Panama-Pacific Dental Congress upon reaching San Francisco, must make provision to secure proper credentials from their state or local dental society, to file with their application. Those not members of any dental society must secure the endorsement of a member of the Executive Committee from the state in which they reside.

### REGISTRATION.

### *The Importance of Registering Early.*

The Bureau of Registration will be located in the Exposition Memorial Auditorium, Grove, Larkin, Hayes and Polk Streets.

A Branch Post Office and Bureau of Information will be established in connection with the Registration Bureau.

Members must register in order to obtain the official program and invitations to entertainments. All are urged to register as soon as they can name their hotels. The Registration Department will be open from 8:30 A. M. to 5:30 P. M. on Monday, August 30, 1915, and these hours will be kept each succeeding day during the Congress, as long as necessary for the accommodation of those wishing to register. Be sure to bring the Membership Card sent you from the San Francisco office when you paid the membership fee.

### HOTEL RESERVATIONS.

Although San Francisco can easily accommodate all those in attendance at the Panama-Pacific Dental Congress, members are urged to make their hotel reservations early. It will be a great comfort upon arriving in San Francisco to go at once to a hotel that is expecting you, rather than to make a round of hotels, finding a number of them completely filled and finally being compelled to take the first lodgings which can be found in a hurried personal search. Reservations may be made through the San Francisco Hotel Bureau, Kearny and Market Sts., S. F., or through the Exposition Tour Service Company, 155 Sutter St., S. F., Cal. Take the reply you receive with you to San Francisco, then on arriving all you will have to do is to go to this hotel and register.

In requesting a hotel reservation be sure to state your full name and home address, the date you expect to reach

San Francisco, the number in your party, the number of rooms required, the rate per day per person, per room—you are willing to pay and the hotal you prefer, if any.

### Mail.

You may have your mail sent to you in care of Panama-Pacific Dental Congress. Exposition Memorial Auditorium, San Francisco, Cal.

### The Following is a Partial List of the Papers to be Given at the Panama-Pacific Dental Congress.

The Evolution and Development of the Mandible. Martin Dewey, D.D.S., M.D., Kansas City, Mo.

Anomalies in Dental Pulp Structures and Their Relation to Clinical Work. Dr. V. A. Latham, Chicago, Ill.

Research on the Resistance of the Red Corpuscles of the Blood of the Human Gums (Normal and Pathological) to the Diluted Salt Solutions. Prof. Anigo Piperno, Rome, Italy.

A Brief Synopsis of a Paper Entitled "An Investigation of Mottled Teeth; an Endemic Affection not Heretofore Known." Dr. Fred. S. McKay, of Colorado, in collaboration with Dr. G. V. Black, Chicago.

The Value of Bacterial Vaccines in the Treatment of Pyorrhea. Dr. Geo. B. Harris, Detroit, Mich.

Radiography in Dentistry and Orthodontia. Drs. Brownlie and Ketcham, Chicago, Ill.

The Etiology of Dental Abscesses, Acute and Chronic. Thos. B. Hartzell, Minneapolis, Minn.

Acidemetric Study of the Saliva and Its Relation to Diet and Caries. John S. Marshall, San Francisco, Cal.

An Investigation of the Character of the Various Dental Cements. Dr. Marcus L. Ward, Ann Arbor, Mich.

Some Refractory Materials used in Dentistry. Dr. Guy Stillman Millberry, San Francisco, Cal.

Report on Dental Clinical Work in the Hospitals, Schools and Prisons in Manila, P. I. Louis Ottofy, D.D.S., Manila, P. I.

The Educational Value of Oral Hygiene in the Army. Dr. Edwin P. Tignor, U. S. Army.

The Agencies in Ohio Co-operating in a General Hygiene Educational Campaign. Dr. Homer C. Brown, Columbus, Ohio.

Hygiene of the Bucco Dental Cavity as an Important Auxiliary for the Prophylaxis of Incipient Bucco-Pulmonary Tuberculosis. Dr. Ernesto A. Dam, Lima, Peru.

Bad Root Canal Work. What Shall We Do About It? Howard R. Raper, D.D.S., Indianapolis, Ind.

Pain—Its Significance and Value as a Diagnostic Symptom—Its Advantages and Disadvantages and the Importance of Its Alleviation and Prevention. Dr. E. T. Loeffler, Ann Arbor, Mich.

Superstitions of Dental Medicine. Dr. Garrett Newkeils, Pasadena, Cal.

Therapeutic and Surgical Treatment of Roots and their Adjacent Tissues. Dr. I. F. Biddle, Pittsburg, Pa.

The Therapeutic of Radicular and Follicular Dental Cysts. Prof. Dr. Rudolph Weish, Vienna, Austria.

A Few Thoughts on the Comparative Anatomy of the Maxillary Sinus—Its Relation to the Teeth, Mandibular Enticulation and Alimentary System. Dr. Matthew H. Cryer, Philadelphia, Pa.

Radium Treatment of Carcenoma. Dr. Oscar Strauss, Milwaukee, Wis.

A Case of Acromegaly. Dr. P. Gaad, Helsingford, Finland.

Etiology and Treatment of Oral Tumors. Dr. Fulton Risdon, Toronto, Canada.

What is the Line of Occlusion. Dr. R. Ottolengui, New York City, N. Y.

An Attempt Toward Automatic Connection. Dr. Sulucana, Spain.

Some Practical Uses in Dental Practice for Tungsten and Molybedenum. Dr. W. A. Price, Cleveland, Ohio.

A Method of Surveying and Mapping the Dental Apparatus. Dr. F. L. Stanton.

The Plantation of Teeth. M. J. Congdon, D.D.S., Berkeley, Cal.

Cavity Preparation for Gold Inlays. Dr. John Conzett, Dubuque, Ia.

The Rescessional Lines of Pulps in Their Relation to Operative Dentistry. Dr. H. G. Chappel, Oakland, Cal.

Operative Procedures in Relation to Dental Caries and Diseases of the Investing Tissues. Arthur D. Black, M.D., D.D.S., Chicago, Ill.

Anoci-Association in Dental Operations. R. H. Reithmuller, Philadelphia, Pa.

Technic in the Treatment of Pulps, Root Canal and Pericpical Area. Dr. M. L. Rhein, New York.

Peridental Anesthesia, Intrasseous Method. Dr. Frank L. Platt, San Francisco, Cal.

The Successful Scientific Treatment of Periodontal Diseases. Dr. T. Sydney Smith, Palo Alto, Cal.

Pyorrhea Alveolaris Showing the Pathological Changes Occurring in the Alveolus Based on Microscopic Observation. Dr. Fred. Hecker, Kansas City, Mo.

The Entameba Buccalis as Seen Through the Microscope, Illustrated by Moving Picture Film and Lantern. H. Page Barley, D.D.S., Los Angeles, Cal.

Impression Material and Impressions. Dr. Geo. H. Neilson, Cleveland, Ohio.

Crown and Bridge. H. J. Goslee, Chicago.

Some Fundamental Things in Dental Prosthesis. Dr. J. Leon Williams, New York.

Some Grave Errors in the Practice of Crown and Bridge Work. Dr. Vincenzo Guerini, Naples, Italy.

Indications and Construction of a Rubber Obturatoa that is Retained Only by the Action of the Soft Tissues. Dr. Calvin S. Case, Chicago, Ill.

Some Educational Topic. Dr. Edw. C. Kiels, Philadelphia, Pa.

The Development of Dental Service in the Navy. Dr. Emory A. Bryant, U. S. N.

Nomenclature. Dr. Arthur D. Black, Chicago, Ill.

Dental Society Organization. Dr. E. S. McCard, Seattle, Wash.

### Clinics.

---

*The following well known men will be among the contributors to the clinics of the Panama-Pacific Dental Congress:*

---

| | |
|---|---|
| Dr. Geo. H. Wilson, | Cleveland, Ohio. |
| Dr. Andrew J. McDonagh, | Toronto, Canada. |
| Dr. J. A. Gardner, | Memphis, Tenn. |
| Dr. J. P. Buckley, | Chicago, Ill. |
| Dr. Fred W. Dethro, | Chicago, Ill. |
| Dr. H. J. Goslee, | Chicago, Ill. |
| Dr. V. H. Jackson, | New York City. |
| Dr. Wm. A. Capon, | Philadelphia, Pa. |
| Dr. Frank M. Castro, | Cleveland, Ohio. |

| | |
|---|---|
| Dr. J. Leon Williams, | London, England. |
| Dr. John D. Conzett, | Dubuque, Iowa. |
| Dr. L. P. Haskell, | Chicago, Ill. |
| Dr. Robin Adair, | Atlanta, Ga. |
| Dr. Thos. B. Hartsell, | Minneapolis, Minn. |
| Dr. Arthur E. Peck, | Minneapolis, Minn. |
| Dr. Edwin R. Kibler, | Indianapolis, Ind. |
| Dr. J. M. Bischoff, | Stevens Pt., Wis. |
| Dr. R. A. Day, | San Francisco, Cal. |
| Dr. C. J. R. Engstrom, | Los Angeles, Cal. |
| Dr. Richard H. Reithmuller, | Philadelphia, Pa. |
| Dr. C. F. Fiset, | Seattle, Wash. |
| Dr. A. H. Ketchan, | Denver, Colo. |
| Dr. James D. McCoy, | Los Angeles, Cal. |
| Dr. Robt. Dunn, | San Francisco, Cal. |
| Dr. Everett M. Heard, | Portland, Ore. |
| Dr. A. F. Merriman, | Oakland, Cal. |
| Dr. Allen Suggett, | San Francisco, Cal. |
| Dr. Fred E. Hart, | San Francisco, Cal. |

---

### The Following Conventions will Meet with the Panama-Pacific Dental Congress.

---

Federation Dentaire Internationale.
Delta Sigma Delta Dental Fraternity.
American Society of Orthodontists.
Southern California Dental Society.
Psi Omega Dental Fraternity National Alumni.
Salt Lake County Dental Society.
National Association of Dental Examiners.
Research Commission of National Dental Association.
House of Delegates National Dental Association.
California State Dental Association.
Utah State Dental Society.

---

### Entertainment at San Francisco.

---

To the Committee of Organization,
Panama-Pacific Dental Congress.

*Gentlemen:* Agreeable to your request we herewith submit service agreed upon for the members of the Panama-Pacific Dental Congress which will hold its meetings in this city from August 29th to September 9th, 1915.

We will furnish rooms with private bath to any of the members of your Congress, their families and friends, commencing Sunday, August 29th, for a period of ten (10) days in either of the following hotels: Clift, Bellevue, Palace, St. Francis, Stewart, Chancellor, Plaza, Somerton, Fielding, Ramona, Kensington, Worth, Wiltshire, Thoma, Glen, Arlington, and other hotels of like character and price respectively, to accommodate all of the members of your Congress, their families and friends who will make reservation within a reasonable period.

It being understood that your members and friends may have choice of hotels at the prices herein quoted up to the limit of the rooms available to us in each hotel respectively, and after such limit has been reached they will be furnished the same accommodations in other hotels of the same price as the hotel selected; all such hotels to be subject to your approval.

We will furnish auto and taxi service from the depot to the hotels on arrival and from the hotels to the depot on departure.

We will transfer all baggage to and from the hotels.

We will give each person ten (10) admissions to the Exposition Grounds. These will be good at any time and may be used by any person.

We will give one auto sight-seeing tour of San Francisco and the Bay Battleship boat criuse on San Francisco Bay, visiting the points of interest and battleships and landing at the Yacht Harbor on the Exposition Grounds.

We will take the wives and daughters of those who take our service on an auto tour, leaving the Clift Hotel at 10 o'clock a. m. on September 2, going out Geary Street to Van Ness Avenue, thence through Van Ness Avenue to the Presidio, through the Presidio, giving them a view of the Golden Gate, also a birdseye view of the Exposition Grounds, State Buildings and Exhibition Palaces from Presidio Heights, thence through Golden Gate Park to the Cliff House, thence along Ocean Drive, Sloat and Junipero Boulevards and the El Camino Real to the Peninsula Hotel at San Mateo. We will furnish and serve a table de hote luncheon to the ladies at the Peninsula Hotel and after luncheon return with them over Portola Drive and Crockett Road, giving them a birdseye view of San Francisco and the Bay from Ashbury Heights and return them to their hotels at about four o'clock p. m.

We will give the wives and daughters of those who take our service a theatre party on Saturday evening, September 4th. Your committee to approve the theatre.

We will include the "Dr. Pague Special Entertainment."

We will have our representative meet all of your special trains at suitable points several hours out of San Francisco, prepared to register your members in their respective hotels, tag and care for their baggage and make all other arrangements necessary for their convenience on arrival.

We will furnish the above specified rooms for ten (10) days, service and entertainment in the following hotels respectively at the prices marked opposite thereof, to-wit:

| | Price of one room when occupied by | | |
|---|---|---|---|
| | 1 Person | 2 Persons | *3 Persons |
| St. Francis Hotel............ | $65.20 | $50.20 | $44.85 |
| Palace and Clift............. | 55.20 | 41.50 | 36.50 |
| Fielding, Ramona and Hotels of same character and price | 51.00 | 36.00 | 32.00 |
| Worth, Kensington and Hotels of same character and price | 37.50 | 30.00 | 27.50 |
| Glen, Arlington and Hotels of same character and price.. | 30.00 | 25.00 | 23.70 |

*Using one double bed and one bed couch.

The prices above set forth are for a full ten (10) days' service and are to be paid as follows:

For each reservation, $10 cash on making reservation, the balance in each case to be paid to us on arrival.

The above payments to be made directly to this company. It is understood that your committee will deliver to us immediately upon receipt, all inquiries and applications for reservations and all payments received on account thereof and that you will cause due publicity to be made of the arrangements for reservations in the regular Dental Publications.

JCH-MS

John C. Hughes,
*General Manager.*

Exposition Service Co.,
155 Sutter St., San Francisco.

We are pleased to recommend the foregoing service and urge you to select the hotel you desire and write immediately for your reservation to the Exposition Tour Service Company at No. 155 Sutter Street, San Francisco, and thereby enable us to place you in the most desirable location.

Make all arrangements to be with us for a record Congress. In packing your trunk DON'T FORGET you will need your heavy, warm clothing and overcoat for evenings and auto trips.

ARTHUR M. FLOOD, D.D.S.,
*Secretary, Committee on Organization.*

